# Microwave Imaging for Parkinson’s Disease Detection: A Phantom-Based Feasibility Study Using Temperature-Controlled Dielectric Variations

**DOI:** 10.3390/s25247562

**Published:** 2025-12-12

**Authors:** Leonardo Cardinali, David O. Rodriguez-Duarte, Jorge A. Tobón Vasquez, Francesca Vipiana, Luis Jofre-Roca

**Affiliations:** 1 Department of Electronics and Telecommunications, Politecnico di Torino, 10129 Torino, Italy; leonardo.cardinali@polito.it (L.C.); david.rodriguez@polito.it (D.O.R.-D.); jorge.tobon@polito.it (J.A.T.V.); 2 Department of Communications and Signal Processing, Universitat Politecnica de Catalunya, 08034 Barcelona, Spain; luis.jofre@upc.edu

**Keywords:** head phantoms, microwave antennas, microwave imaging, Parkinson’s disease

## Abstract

Parkinson’s disease (PD) is characterized by pathological changes in the substantia nigra, which in its early stages may manifest as structural and functional asymmetries between the two hemispheres. Microwave imaging has recently emerged as a promising non-invasive tool to detect subtle dielectric variations. In the context of Parkinson’s disease, such contrasts are expected to arise from the underlying physiological alterations in brain tissue, although their magnitude has not yet been fully characterized. In this work, we investigate the feasibility of differential microwave imaging, where detection is based on permittivity contrasts, through a controlled phantom study. A simple two-dimensional head phantom was constructed using a 3D-printed cylindrical container filled with water, incorporating a Teflon tube to represent the substantia nigra. The tube was filled with hot water, whose gradual cooling emulated small dielectric changes. Since the dielectric properties of water vary linearly with temperature over 0.5–3 GHz, we first validated this dependence through both numerical analysis and experimental measurements. Four antennas were then employed in a differential imaging configuration, with image reconstruction performed via the multi-frequency bi-focusing algorithm. The results show that the system can successfully detect a dielectric contrast corresponding to a temperature variation as small as 0.4 °C, equivalent to approximately 0.17% in relative permittivity. While the exact dielectric changes associated with PD remain to be determined, these results demonstrate that the proposed approach is sensitive to very small contrasts, supporting the potential of differential microwave imaging as a candidate tool for future investigations into Parkinson’s disease detection.

## 1. Introduction

### 1.1. Overview of Parkinson’s Disease

Parkinson’s disease (PD) is one of the fastest-growing neurological disorders worldwide due to aging populations. Its increasing prevalence, chronic progressive course, and long-term management needs create a substantial socio-economic burden on patients, caregivers, and health systems. PD is a progressive neurodegenerative disorder characterized by the loss of dopaminergic neurons in the substantia nigra pars compacta (SNpc), a dopaminergic nucleus of the midbrain and a key component of the basal ganglia. Its neurons provide dopaminergic input to the striatum, modulating basal ganglia pathways that control voluntary movement, reward, and habit learning. This neuronal loss is associated with the accumulation of misfolded alpha-synuclein, which aggregates into Lewy bodies and contributes to dopaminergic neurodegeneration. Neuropathological changes in PD are normally asymmetric in the early stages, with one hemisphere showing a greater dopaminergic loss than the other [1,2]. Clinically, PD presents with both motor and non-motor symptoms. Motor symptoms include bradykinesia, resting tremor, rigidity, and postural instability. Non-motor symptoms are various, including cognitive impairment, autonomic dysfunction, and mood disorders, often preceding motor onset by years [3]. The disease usually arises in later adulthood, but there is high variability in its progression.

Current therapeutic strategies for PD consist primarily of dopaminergic replacement therapies, such as levodopa and dopamine agonists. These treatments provide substantial relief of motor symptoms but do not halt or reverse neurodegeneration, and their long-term use is complicated by fluctuations and dyskinesias [4]. Adjunctive therapies, including monoamine oxidase-B, catechol-O-methyltransferase inhibitors [5], and deep brain stimulation (DBS) [6], can further improve motor control, particularly in advanced stages. However, all available interventions are considerably more effective when initiated early. Consequently, timely detection of PD is critical to maximize therapeutic benefit, maintain quality of life, and potentially extend the therapeutic window for emerging disease-modifying approaches currently under investigation [7].

### 1.2. Diagnostic Techniques

PD diagnosis remains primarily clinical, based on history and neurological examination supported by validated rating scales. The Movement Disorder Society-sponsored revision of the Unified Parkinson’s Disease Rating Scale (MDS-UPDRS) is currently the most widely used clinical scale to assess Parkinson’s disease symptoms and progression [8]. While clinical expertise often enables accurate diagnosis of Parkinson’s disease, overlapping symptoms with atypical parkinsonian syndromes and the diagnostic uncertainty in early stages remain common challenges [9].

Neuroimaging and molecular tests are widely used as adjuncts. Dopamine transporter single-photon emission computed tomography (DaT-SPECT) and photon emission tomography (PET) imaging assess presynaptic dopaminergic integrity and improve diagnostic confidence in clinically uncertain cases. Structural magnetic resonance imaging (MRI) contributes to excluding alternative causes, and advanced MRI techniques (e.g., diffusion MRI, neuromelanin imaging) add pathophysiological insight [10,11,12]. The development of biomarkers for Parkinson’s disease in cerebrospinal fluid (CSF) and blood is an active area of research, with particular focus on alpha-synuclein species (total, phosphorylated, and oligomeric forms), neurofilament light chain (NfL), and other candidates such as tau proteins, amyloid-beta peptides, and inflammatory markers [13,14,15]. Despite these advances, many of the imaging and biomarker methods are costly, not universally available, or still under validation.

While cerebrospinal fluid and plasma biomarkers hold promise, their translation into early diagnostic tools for Parkinson’s disease is hindered by invasiveness, variability, and lack of disease specificity [16]. Recent years have seen a proliferation of digital biomarkers and wearable sensor approaches for PD that quantify gait, tremor, bradykinesia, and other motor features objectively in home or clinical settings. These approaches include inertial measurement units (IMUs) [17], smartphone sensors [18], and other wearable devices [19,20]. These technologies are promising for early detection, continuous monitoring, and therapy optimization, but they typically capture peripheral motor manifestations rather than direct central nervous system pathology.

MRI can detect changes in neuromelanin content and iron accumulation in the SNpc, as well as microstructural alterations using diffusion-based metrics [21]. PET imaging is used to investigate dopaminergic dysfunction before symptoms [22], while DaT-SPECT, similarly, captures presynaptic dopaminergic deficiency [23], leading to the possibility of early PD detection. These modalities have high clinical value but are expensive and not ideal for broad screening. Hence, there is a need for accessible, non-ionizing, low-cost imaging modalities that can complement existing approaches, especially for large-scale screening.

### 1.3. Microwave Imaging for Parkinson’s Disease

Microwave imaging (MWI) is a non-ionizing technique that is sensitive to the dielectric properties (relative permittivity and conductivity) of biological tissues; it has been actively investigated for applications such as breast imaging [24,25,26,27,28], stroke detection [29,30,31], brain tumor detection [32,33], and neurodegenerative diseases such as Alzheimer’s disease [34,35,36]. Functional microwave imaging using modulated bio-tags to mimic neural activity has also been investigated for detecting changes associated with Parkinson’s disease in controlled head phantom experiments [37]. MWI systems are portable, safe, and relatively low-cost, and they have substantially improved image quality and sensitivity in recent years due to advances in antenna design, inverse algorithms, machine learning reconstructions [38], and related fields such as metasurfaces, which are able to improve the coupling efficiency and sensitivity of microwave-based brain imaging systems [39]. These properties position MWI as an attractive complementary modality for neurological applications, where subtle tissue property changes may carry diagnostic information. Applied to PD, a differential imaging strategy could exploit early hemispheric asymmetries or small localized dielectric contrasts potentially associated with pathology; however, key unknowns remain: the magnitude and spatial extent of dielectric changes in PD tissue are not yet well established, and resolving small, deep structures such as the SNpc is challenging due to size and low contrast. Therefore, feasibility studies that quantify the minimum detectable contrast and evaluate algorithmic performance in realistic phantom environments are essential first steps.

**Figure 1:**
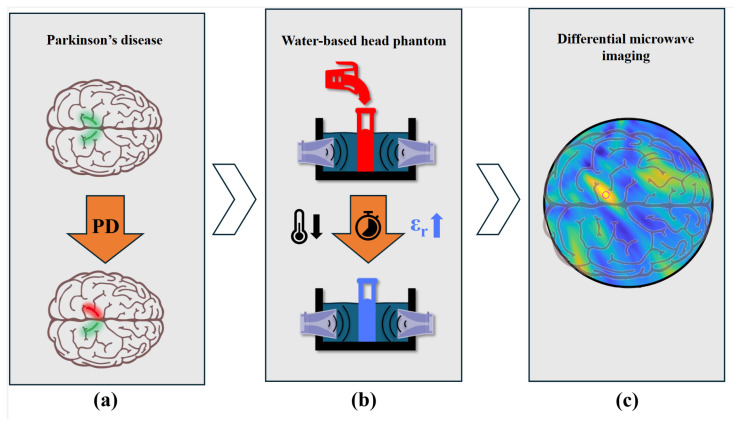
Artist’s view. (**a**) Parkinson’s disease induces structural and dielectric asymmetries in the substantia nigra during the early onset of the illness. (**b**) Measurements were performed using a water-based head phantom, where the dielectric contrast of the target was achieved through controlled temperature variation of the water. (**c**) Images were reconstructed from the measured data, and a sensitivity analysis of the system was conducted.

As depicted in Figure 1, this paper presents a controlled phantom study designed to quantify the sensitivity limits of a differential MWI system for small permittivity differences representative of subtle pathological changes. These changes were obtained through controlled temperature variations of deionized water. The inspiration for using this strategy came from [40], where MWI was explored for remote thermal sensing. This study employed a 2D head phantom with a temperature-controlled inclusion, exploiting the linear dependence of water’s permittivity on temperature across 0.5–2 GHz, and reconstructing differential images with a four-antenna multi-frequency bi-focusing algorithm. The objective was to determine the smallest relative permittivity change detectable by the system, providing a necessary experimental benchmark for subsequent investigations. Preliminary results of this work have been recently presented in [41].

The rest of this article is organized as follows: Section 2 presents the numerical and experimental analysis of the water’s dielectric properties and discusses its suitability for use in dielectric phantoms. Section 3 describes the overall microwave imaging system, including the antenna configuration and the algorithm employed to obtain the reconstructed images. Section 4 details the design and fabrication of the phantom, along with the measurement protocol. The experimental results are analyzed and discussed in Section 5, while Section 6 summarizes the main findings and outlines future research directions.

## 2. Temperature-Dependent Dielectric Characterization

Microwave-based sensing and imaging rely on accurate knowledge of the dielectric properties of biological tissues or tissue-mimicking materials. Deionized water is commonly used in anthropomorphic phantom designs due to its well-characterized dielectric behavior. Its dielectric properties (i.e., the relative permittivity, $\varepsilon_{r}$, and the conductivity, *σ*) depend on temperature, *T*, and frequency, *f*. Our goal here is to exploit this relationship to obtain controlled and repeatable differential dielectric variations in phantoms without modifying their geometry or composition.

**Figure 2:**
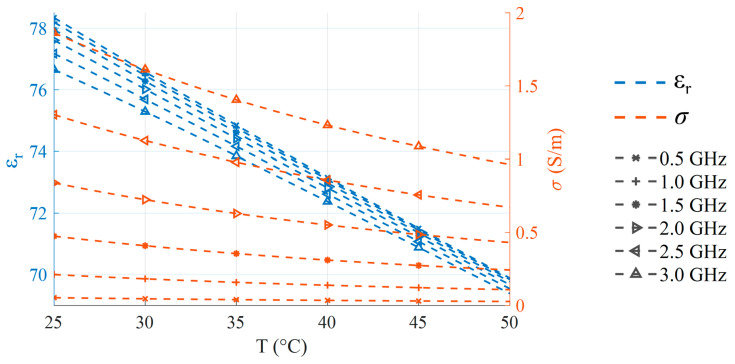
Temperature-dependent values of $\varepsilon_{r}$ (dashed blue) and σ (dashed orange) calculated between 25 °C and 50 °C at key frequency points using the Debye model. For both properties, all of the considered frequencies show highly linear behavior ($R^{2}>0.985$).

The complex permittivity of water, ε(f,T), can be described by the Debye relaxation model:

(1) $$\varepsilon \left(f,T\right)=\varepsilon_{\infty }\left(T\right)+\frac{\varepsilon_{s}\left(T\right)-\varepsilon_{\infty }\left(T\right)}{1+j2\pi f\tau (T)},$$

where $\varepsilon_{s}$ is the static permittivity, $\varepsilon_{\infty }$ is the permittivity at infinite frequency, and τ is the relaxation time. Each of these parameters exhibits a well-defined temperature dependence, which is reported in [42]. Based on this model, the values of $\varepsilon_{r}$ and σ in the temperature range from 25 °C to 50 °C were calculated for the frequency points of 0.5 GHz, 1 GHz, 1.5 GHz, 2 GHz, 2.5 GHz, and 3 GHz, as shown in Figure 2. Both $\varepsilon_{r}$ and σ exhibit a linear behavior with respect to temperature in this temperature range for these frequencies, and this linear behavior can be quantified using the coefficient of determination $R^{2}$ [43], defined as follows:

(2) $$R^{2}=1-\frac{SS_{\mathrm{res}}}{SS_{\mathrm{tot}}},$$

where the residual sum of squares $SS_{\mathrm{res}}$ and the total sum of squares $SS_{\mathrm{tot}}$ are given by

(3) $$SS_{\mathrm{res}}=\sum_{i}{\left(y_{i}-{\hat{y}}_{i}\right)}^{2},\;SS_{\mathrm{tot}}=\sum_{i}{\left(y_{i}-\bar{y}\right)}^{2}$$

Here $y_{i}$ represents the *i*-th measured value, ${\hat{y}}_{i}$ is the corresponding value estimated by the linear model, and $\bar{y}$ is the mean of all measured values.

For all considered frequencies, both $\varepsilon_{r}$ and σ exhibit a calculated $R^{2}>0.985$ over the temperature range, which indicates behavior close to a linear trend (i.e., $R^{2}=1$).

To validate this theoretical model, measurements were carried out on deionized water samples. The liquid was first heated to 36.5 °C, and then the dielectric properties were recorded during the cooling phase until the liquid reached a temperature of 26.0 °C. An RS PRO RS1710 digital thermometer (RS PRO, Corby, UK) with a resolution of 0.1 °C was used to track the temperature. At each 0.1 °C interval, the complex permittivity was measured using a coaxial probe connected to a Keysight N9918A vector network analyzer (VNA) (Keysight Technologies, Santa Rosa, CA, USA) [44]; the software used to retrieve the measurements and calculate $\varepsilon_{r}$ and σ was the Keysight Materials Measurement Suite software version 20.0.24092501 [45]. The relative sensitivities of the relative permittivity, $\eta_{\varepsilon_{r}}$ and of conductivity, $\eta_{\sigma }$, for a 0.1 °C temperature change were computed as follows:

(4) $$\eta_{\sigma }=\frac{\sigma \left(26\;{}^{\circ}C\right)-\sigma \left(36.5\;{}^{\circ}C\right)}{n_{T}\cdot \sigma \left(26\;{}^{\circ}C\right)},\;\eta_{\varepsilon_{r}}=\frac{\varepsilon_{r}\left(26\;{}^{\circ}C\right)-\varepsilon_{r}\left(36.5\;{}^{\circ}C\right)}{n_{T}\cdot \varepsilon_{r}\left(26\;{}^{\circ}C\right)},$$

where $n_{T}$ is the number of temperature steps (0.1 °C increments, so $n_{T}=105$). Dividing the total change by $n_{T}$ converts the overall variation into a per-step change. The normalization was performed using the final value at 26 °C. This choice provides a stable reference for the relative change, as the dielectric properties decrease with temperature, and yields a dimensionless sensitivity that represents the fractional change relative to the lower-temperature state.

**Figure 3:**
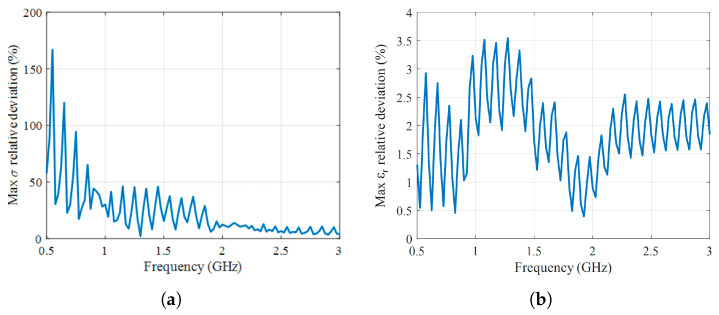
Maximum relative deviation depending on frequency between the predictions of the Debye model and the measured dielectric properties: (**a**) Maximum relative deviation of conductivity. (**b**) Maximum relative deviation of relative permittivity.

The maximum relative deviations as a function of frequency, computed over all temperature measurements, are reported in Figure 3 for both $\varepsilon_{r}$ and σ. For $\varepsilon_{r}$, the largest deviation is 3.56 % at 1.275 GHz, indicating that the measured permittivity closely follows the Debye prediction across the entire band. In contrast, the relative deviation of σ is substantially higher at the lower end of the frequency range and progressively decreases toward higher frequencies. This behavior arises because the intrinsic conductivity of water is very small at low frequencies, approaching 0 S/m, so even a small absolute measurement error leads to a large relative deviation when compared to the theoretical value. Moreover, the imaginary part of the complex permittivity extracted from the measured scattering parameters is affected by the VNA phase noise and by inversion-related uncertainties, which become dominant when σ approaches zero. At higher frequencies, where water increases in conductivity, the same absolute error results in a much smaller relative difference, around 7 %. There are systematic deviations observed between the measured dielectric properties and the Debye model that can be attributed to several factors. Among these are VNA-related uncertainties, particularly phase noise and drift, which primarily affect the imaginary part of the permittivity and, therefore, the reconstructed conductivity. Other factors include non-ideal conditions such as finite water purity, residual ionic content, and temperature measurement inaccuracies, all of which introduce consistent offsets. Moreover, small calibration imperfections or mismatches at the probe–medium interface introduce systematic biases.

**Figure 4:**
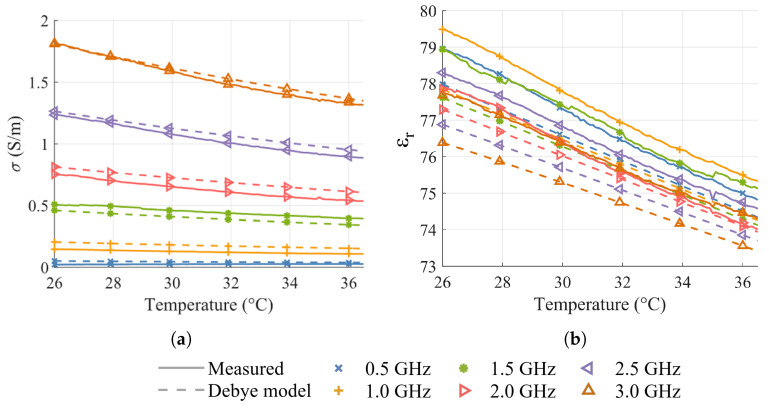
Dielectric properties of deionized water as a function of temperature: (**a**) Conductivity. (**b**) Relative permittivity.

Despite these inaccuracies, the measurements exhibit the expected linear dependence on temperature at every analyzed frequency. The experimental results reported in Figure 4 demonstrate a linear dependence of both $\varepsilon_{r}$ and σ on temperature at the examined frequency points. The coefficient of determination $R^{2}$ exceeds 0.99 for all $\varepsilon_{r}$ measurements and remains above 0.98 for all σ measurements, with the sole exception of 0.5 GHz, where $R^{2}=0.865$. This reduction in linearity at the lowest frequency is consistent with the previously discussed challenges associated with conductivity reconstruction when σ is intrinsically small, making the measurement particularly sensitive to noise and uncertainties. The measured relative sensitivities, summarized in Table 1 and Table 2, further indicate that the dielectric properties can be finely controlled, with σ varying by about $4.7\times 10^{-4}\%$ and $\varepsilon_{r}$ varying by about $4.2\times 10^{-2}\%$ for each 0.1 °C step.

Water temperature control provides a simple and precise method for modulating the dielectric properties of water-based phantoms. This method allows linear and repeatable changes in permittivity and conductivity without altering the phantom composition. It is particularly useful for differential measurements aimed at evaluating microwave sensing and imaging systems. It is important to note that water does not replicate the absolute dielectric properties of brain tissue; therefore, the temperature-induced variations studied here should not be interpreted as direct physiological equivalents. Instead, the controlled modulation of $\varepsilon_{r}$ and σ provides a well-defined and repeatable way to determine the minimum relative contrast that the system is able to resolve. The sensitivity obtained from water measurements should be understood as a threshold expressed in terms of percentage change, rather than absolute permittivity values. When comparing these relative thresholds with the small dielectric variations expected in biological tissue, the results remain indicative of the system’s potential detection capability.

Hence, in this study, water at different controlled temperatures was employed to obtain a phantom that gradually varies its dielectric properties in a target location. By acquiring the corresponding microwave responses, the developed imaging system was tested for its sensitivity to small variations in permittivity. This controlled scenario enables a preliminary assessment of the system’s ability to detect subtle dielectric contrasts that may be associated with early pathological changes, such as those occurring in the brain during the onset of Parkinson’s disease.

## 3. Microwave Imaging System

### 3.1. System Overview

The microwave imaging system, inspired by [46,47], consisted of four broadband antennas connected to a Rohde & Schwarz ZNA26 four-port VNA (Rohde & Schwarz, Munich, Germany) [48], allowing for the acquisition of *S*-parameters without requiring a switching matrix. The frequency range was set between 0.5 and 3 GHz, with 201 frequency points, an input power level of 0 dBm, and an intermediate-frequency (IF) bandwidth of 10 kHz. This frequency range represents a trade-off between penetration and resolution: lower frequencies ensure deeper penetration into the phantom, while higher frequencies provide improved spatial resolution due to the shorter wavelength. Using 201 frequency points allows for a sufficiently fine sampling to ensure stable inversion while keeping the overall acquisition time manageable. The input power level of 0 dBm avoids nonlinearities and guarantees a suitable signal-to-noise ratio (SNR). Additionally, the 10 kHz IF bandwidth represents an appropriate filter setting, which increases the measurement stability and SNR at the cost of a slightly longer acquisition time, in the order of tenths of a second. These parameters were selected according to the operating bandwidth and sensitivity characteristics of the antennas used in this study.

**Figure 5:**
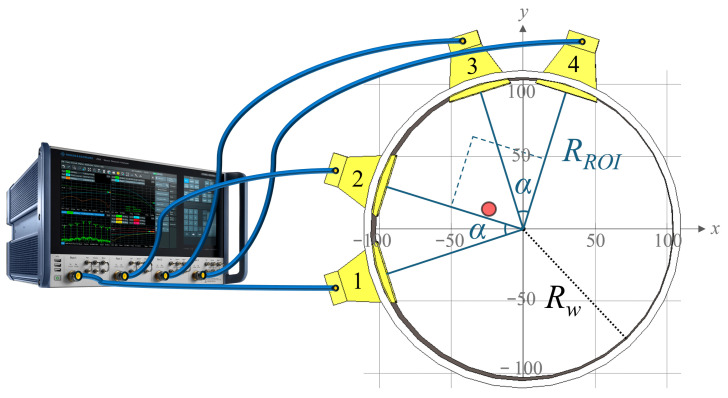
System scheme: (**left**) Vector network analyzer. (**right**) Antenna arrangement inside the phantom walls. The target location for the experiment is shown as a red circle. The values displayed in the grid are in mm.

The four antennas were positioned based on the analysis conducted in [46] for brain imaging applications. The antennas were positioned on a circular array with a radius $R_{ROI}=100$ mm, measured from the antenna apertures, approximating the dimensions of an average human head. As shown in Figure 5, antennas 1 and 2 formed a pair separated by an angular distance of α = 35°, while antennas 3 and 4 formed a second, identical pair. The two pairs were arranged orthogonally: antenna 1 was positioned 90° apart from antenna 3, and antenna 2 from antenna 4. This configuration provides balanced angular coverage, ensuring that antennas do not overlap, while focusing the overall sensitivity region at the center of the circle—corresponding to the target region of interest (ROI), where the SNpc is located within the brain. The ROI was defined as a circular area with a radius of 100 mm lying in the transverse plane shared by all four antennas. Its center coincided with the central axis of the cylindrical phantom, whose inner radius $R_{w}$ was 105 mm. The slightly larger phantom radius accommodates the mechanical support for the antennas, ensuring that their apertures lie exactly on the outer boundary of the ROI. The antennas were mounted so that their pointing direction would intersect the transverse plane at mid-height of the phantom (50 mm from the bottom) and, therefore, protrude slightly inside the phantom walls to achieve the correct positioning. The phantom details are discussed in Section 4. The geometric arrangement was optimized to maximize the overlap between the individual antenna beam patterns and the ROI, enhancing spatial focusing and minimizing the influence of boundary reflections.

### 3.2. Antennas

**Figure 6:**
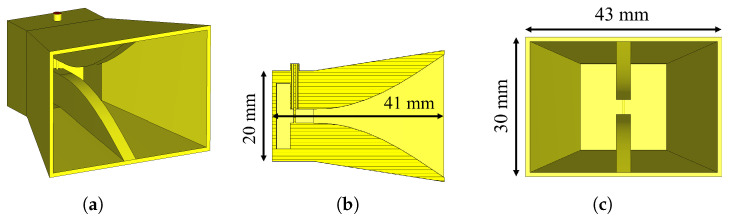
Extended gap ridge horn antenna used for the imaging system; the main antenna dimensions are shown. The complete characterization of the antenna is described in [49]. (**a**) Three-dimensional view. (**b**) Side view with cutting plane. (**c**) Front view.

The imaging system employed four extended gap ridge horn (EGRH) antennas, designed for broadband operation and stable radiation characteristics across a wide frequency range, as shown in Figure 6. These antennas are general-purpose propagation probes for biomedical measurements, described in [49], with a band of interest between 0.5 GHz and 3 GHz when matched to biological tissues. They exhibit unidirectional convenient gain, compact size, and low cross-polarization, making them well suited for near-field microwave imaging applications.

The original antenna design is optimized for a coupling medium with a relative permittivity of $\varepsilon_{r}\approx 50$, providing a nominal Standing Wave Ratio (SWR) of less than 2 ($|S_{11}|\;<-10$ dB) across the 0.5 GHz to 1.5 GHz band, and an SWR lower than 3.5 ($|S_{11}|\;<-5$ dB) across the 1.5 GHz to 3 GHz band. However, in our experimental setup, the antennas radiated into deionized water ($\varepsilon_{r}\approx$ 74–79). This difference introduced an expected slight spectral shift in the operating band compared to the nominal design.

**Figure 7:**
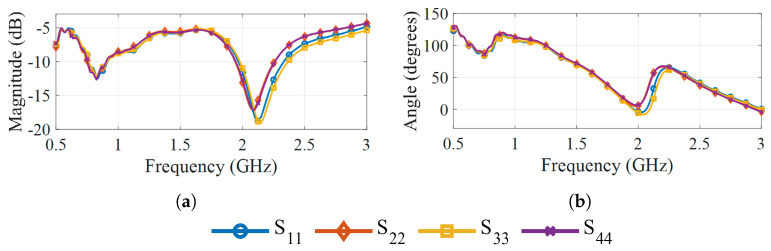
Reflection parameters of the antennas in water at 25 °C: (**a**) Magnitude. (**b**) Phase.

**Figure 8:**
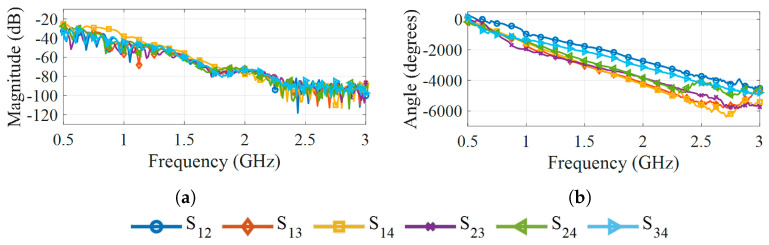
Transmission parameters of the antennas in water at 25 °C: (**a**) Magnitude. (**b**) Phase.

The measured antenna reflection parameters ($S_{ii}$) are shown in Figure 7, and the transmission parameters ($S_{ij}$) in Figure 8. In both cases, the antennas operate while radiating into deionized water, and their disposition is the same as shown in Figure 5. The magnitude of the reflection coefficients, shown in Figure 7a, remains below −5 dB throughout the entire band and stays under −10 dB between 0.75 GHz and 0.9 GHz, as well as for frequencies between 2 GHz and 2.3 GHz. For frequencies above 2.3 GHz, the transmission magnitude drops below −80 dB, as visible in Figure 8a, indicating that only a negligible fraction of the transmitted energy reaches the receiving antennas at these frequencies. This observation suggests that frequencies above 2.3 GHz contribute little useful information for image reconstruction. The corresponding phase responses of the transmission parameters in Figure 8b exhibit consistent trends under 2.3 GHz but show a noisier behavior beyond that frequency.

**Figure 9:**
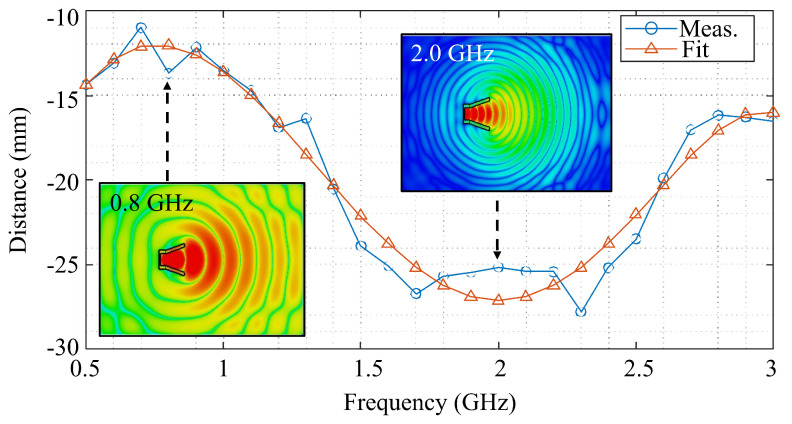
Phase center reconstruction: The blue line with circles represents the measured distance of the phase center from the aperture; the red line with triangles represents the fit polynomial. Top views of the electric field distributions that were used to calculate the phase center are shown for 0.8 GHz and 2.0 GHz.

In antenna theory, the phase center is conceptually defined as the point from which an antenna radiates as if it were an ideal point source (i.e., from which equiphase surfaces emanate). Locating the phase center is crucial to ensure accurate path length estimation and phase alignment, which directly affect the quality of the reconstructed image. In order to do that, full-wave simulations are performed using a frequency-domain solver, and the electromagnetic field distribution emitted by the antenna is analyzed for each frequency point between 0.5 GHz and 3 GHz, with a 0.1 GHz step. In the simulation, the antenna was immersed in a homogeneous water medium at 25 °C, corresponding to the same medium used in the experiments. The dielectric behavior of water followed the Debye model described in Section 2. The phase center was evaluated at each frequency point to account for the frequency-dependent radiation characteristics. Although the antennas operated in the near field and the wavefronts were not perfectly spherical, the horn radiation retained sufficient symmetry such that the intersection with the transverse plane formed an approximately circular equiphase pattern in the region where the ROI would be. The centroid was calculated over the points lying on the same equiphase surface in the transverse plane, ensuring that only the significant portion of the wavefront contributed to the determination of the phase center. The simulation domain was defined as a rectangular box extending 200 mm in front of the antenna’s aperture, 100 mm behind the antenna’s back wall, and 200 mm in each of the two transverse directions. This configuration reproduced the spatial region covered by the experimental ROI. The field within this volume—specifically, in the transverse plane intercepting the main beam—was used to extract the centroid of the equiphase contour and determine the phase center. To evaluate the sensitivity of the computed phase center to modeling errors, the simulation domain boundaries were varied by ±5 mm in each direction. The resulting centroid position remained unchanged across all frequency points, demonstrating that the calculated phase center is robust with respect to small variations in the simulation setup. The measured distance from the aperture and the corresponding fifth-order polynomial fit describing its frequency dependence are shown in Figure 9. Ideally, the phase center’s location should evolve smoothly with frequency, but the simulated values show small irregularities across the band. These fluctuations are due to subtle interactions between the radiated field and the absorbing boundaries of the computational domain, which slightly affect the shape of the retrieved equiphase contour. At 2 GHz this sensitivity is marginally higher, resulting in the more noticeable deviation seen in the plot, even though the antenna performance at that frequency remains optimal. This model enables precise compensation during image reconstruction, ensuring accurate focusing in multi-frequency measurements.

### 3.3. Reconstruction Algorithm

To elaborate the *S*-parameters measured with the system described in Section 3.1, we used the multi-frequency bi-focusing (MFBF) algorithm, which is a quasi–real-time reconstruction algorithm demonstrating robust and reliable imaging performance, introduced in [50]. In the present study, the objective was to reconstruct a two-dimensional image of a head phantom. To generate the image, the dimensions of the ROI and the number of pixels, each corresponding to a focal point within the ROI, must be defined. The ROI adopted in this work is a circle with a radius of 100 mm, centered at the midpoint of the antenna array, thereby encompassing the entire region inside the phantom. Each pixel corresponds to 1 mm^2^, providing a good compromise between computational time and spatial resolution.

The image reconstruction was performed on a pixel-by-pixel basis using the scattering parameters measured by the VNA. The image was formed by analyzing the difference between the measurement under test and a background. The reference background is the healthy condition, when the internal target is at 40.5 °C. Subsequently, as the target naturally cools down over time, the permittivity increases, mimicking a pathological condition. For each temperature step, the differential scattering matrix Δ*S* is computed as follows:

(5) $$\Delta S=S_{background}-S_{target},$$

where $S_{background}$ corresponds to the scattering matrix collected at the reference (healthy) temperature, and $S_{target}$ corresponds to the scattering matrix collected at the temperature under test (pathological). The resulting differential data were used as inputs for the image reconstruction algorithm, allowing the visualization of permittivity variations with respect to the healthy baseline. Because early-stage PD affects one hemisphere more than the other, the contralateral hemisphere can be used as the background, allowing the detection of asymmetries in dielectric properties within the brain.

Each pixel corresponds to a spatial location, referred to as a focal point $\left(x_{f_{p}},y_{f_{p}}\right)$, for which the intensity is computed as follows:

(6) $$I\left(x_{f_{p}},y_{f_{p}}\right)=\sum_{f}\sum_{T_{i}}\sum_{R_{j}}\frac{\Delta S_{T_{i}R_{j}}\left(f\right)}{k^{2}}\cdot e^{jk\rho_{R_{j},f_{p}}}\cdot e^{jk\rho_{T_{i},f_{p}}},$$

where $I(x_{f_{p}},y_{f_{p}})$ represents the reconstructed intensity at the focal point $\left(x_{f_{p}},y_{f_{p}}\right)$, *f* is the operating frequency, $\Delta S_{T_{i}R_{j}}\left(f\right)$ is the differential transmission scattering parameter between the transmitting antenna $T_{i}$ and the receiving antenna $R_{j}$, and k(f) is the wavenumber of the electromagnetic wave in the background medium (in this case, water) at frequency *f*, given by

(7) $$k\left(f\right)=\frac{2\pi f\sqrt{\varepsilon_{r}\left(f\right)}}{c},$$

where $\varepsilon_{r}\left(f\right)$ is the relative permittivity of the medium, and *c* is the speed of light in a vacuum. The terms $\rho_{T_{i},f_{p}}$ and $\rho_{R_{j},f_{p}}$ represent the Euclidean distances between the focal point and the transmitting and receiving antennas, respectively, defined as follows:

(8) $$\rho_{R_{j},f_{p}}=\sqrt{{\left(x_{R_{j}}-x_{f_{p}}\right)}^{2}+{\left(y_{R_{j}}-y_{f_{p}}\right)}^{2}},\;\rho_{T_{i},f_{p}}=\sqrt{{\left(x_{T_{i}}-x_{f_{p}}\right)}^{2}+{\left(y_{T_{i}}-y_{f_{p}}\right)}^{2}}$$

Each reconstructed pixel intensity provides a dimensionless indicator of the local dielectric contrast, proportional to the variation in the dielectric properties with respect to the background reference.

## 4. Experimental Assessment

### 4.1. The Phantom

A custom 3D-printed head phantom was designed and fabricated to experimentally assess the system’s sensitivity to dielectric variations mimicking the onset of Parkinson’s disease. The phantom has two main simplifications compared to a realistic head: First, it consists of a homogeneous water volume rather than a multilayer structure. As a result, the impedance discontinuities and attenuation that would normally affect the fields inside the head are not present. Second, the phantom adopts a simplified 2D cylindrical geometry, in which both the head and the localized perturbation mimicking the SNpc are modeled as cylindrical regions rather than three-dimensional structures, thereby removing the spatial complexity that would affect field propagation in vivo. Consequently, the detectability assessment obtained with this model represents an optimistic scenario relative to the actual anatomical conditions. The design provides a simplified and controllable environment where localized dielectric perturbations can be precisely introduced and monitored. This approach allows focusing on the detectability of small variations in permittivity occurring at the position of a specific brain region: the SNpc.

**Figure 10:**
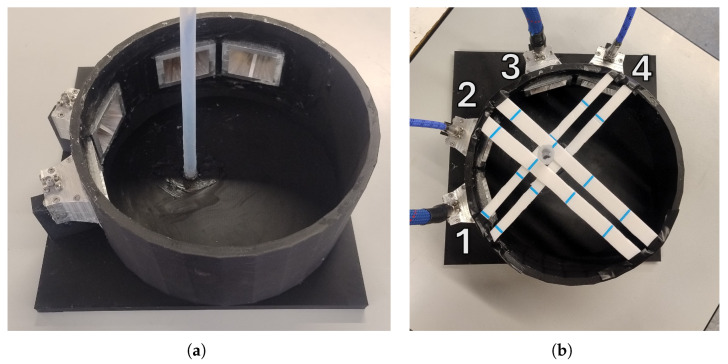
Water-based printed phantom used for experimental validation: (**a**) The phantom, empty and disconnected from the VNA. (**b**) The phantom, filled and connected to the VNA. Plastic strips were added to ensure higher stability of the Teflon tube.

The phantom, as shown in Figure 10, was printed in polylactic acid (PLA) using an Original Prusa XL 3D printer (Prusa Research, Prague, Czech Republic) with fused deposition modeling (FDM) and consisted of a cylindrical structure with an internal radius of 105 mm, a thickness of 5 mm, and a height of 100 mm. Four apertures were integrated into the cylinder wall to accommodate the antennas, while dedicated supports ensured their stable positioning and maintained each antenna’s aperture precisely 100 mm from the central axis of the phantom. The internal cavity was filled with deionized water at room temperature, which also extended into the antenna slots, providing an effective dielectric matching medium and ensuring a stable measurement environment.

The antennas were externally coated with a protective conformal lacquer (Electrolube, Ashby de la Zouch, UK), and the apertures were sealed with silicone after antenna insertion to prevent any water leakage from the phantom. At the position corresponding to the SNpc, where early PD typically exhibits lateralized degeneration [51], a Teflon tube (Sigma-Aldrich, St. Louis, MO, USA) was inserted and fixed to the bottom of the phantom using silicone. The tube had an internal diameter of 8 mm, chosen to replicate the two-dimensional scale of the SNpc region in the brain, and a thickness of 1 mm. It was placed off-center relative to the phantom’s central axis, in order to emulate the position of the SNpc’s lateralized expected dielectric change due to early PD. Referencing the coordinate system in Figure 5, the center of the cylinder was located at x=−25 mm and y=10 mm. The tube was filled with deionized water initially heated to 40.5 °C, representing the “healthy” dielectric state. As the water gradually cooled, its dielectric constant increased, thereby simulating the pathological dielectric change associated with neuronal loss and glial scar formation in Parkinson’s disease. The complete phantom and antennas system is shown in Figure 10.

### 4.2. Thermal Analysis

The phantom was a water-filled cylinder (internal radius 105 mm, height 100 mm; volume ≈3.46 L). The heated target was a tube (internal radius 4 mm, wall thickness 1 mm, length 100 mm) containing approximately 7.8 g of water, initially at 40.5 °C. The thermal capacity of a water volume *m* is computed as follows:

(9) $$C=m\;c_{p},$$

where *m* is the mass and $c_{p}=4186\;\;J/\left(\mathrm{kg}\cdot \;{}^{\circ}C\right)$ is the specific heat of water. Thus, the bulk water in the phantom (mass ≈ 3.46 kg) has a thermal capacity of $\approx 1.45\times 10^{4}\;\;J/{}^{\circ}C$, while the water inside the target (mass ≈0.0078 kg) has a thermal capacity of ≈33 J/°C. The heat *Q* released by the target during its cooling from 40.5 °C to 36 °C (Δ*T* = 4.5 °C) is computed as follows:

(10) $$Q=C_{\mathrm{target}}\cdot \Delta T\approx 148\;\;J,$$

where $C_{\mathrm{target}}$ is the thermal capacity of the target. Assuming conservatively that all of this heat is transferred to the remaining water outside the target, the corresponding temperature increase is as follows:

(11) $$\Delta T_{\mathrm{phantom}}=\frac{Q}{C_{\mathrm{phantom}}}\approx 0.01\;{}^{\circ}C,$$

where $C_{\mathrm{phantom}}$ is the thermal capacity of the phantom. Moreover, the characteristic convective cooling time constant *τ* of a water volume *V* is given by

(12) $$\tau =\frac{\rho c_{p}V}{hA},$$

where ρ is the water density, $c_{p}$ is the specific heat of water, *V* is the considered water volume, *h* is the air–water convective coefficient, and *A* is the surface area. For an open-topped water container undergoing natural convection, a realistic range of *h* associated with laboratory environments is 5 to 10 $W\;m^{-2}\;K^{-1}$. The corresponding τ range for these values is between 6 and 3 h, much larger than the total duration of the experiment (237 s). Therefore, on the timescale of the measurements, both the ambient temperature and the bulk temperature of the phantom remain effectively constant, and any thermal variation in the surrounding water is negligible compared to the 0.1 °C resolution used in the experiment.

### 4.3. Measurement Protocol

The measurement protocol began with the calibration, performed at the antenna ports, of the four-port VNA connected to the antenna system. The calibration was performed using the Rhode & Schwarz ZN-Z151 calibration unit. Then, room-temperature deionized water was introduced into the phantom body, and heated deionized water was injected into the Teflon tube acting as the target. Once the system was stabilized, the temperature inside the Teflon tube was continuously monitored with a digital thermometer.

The scattering parameters of the four antennas were recorded each time the water temperature decreased by 0.1 °C, covering the range from 40.5 °C down to 36 °C: a range in which the Debye model remains valid and predicts a linear variation in the dielectric properties with temperature.

The total duration to collect all 46 samples was 237 s for a total of 45 intervals between measurements. The measurement times were extracted from the *S*-parameter files saved by the VNA, with a precision of one second. The average time between consecutive acquisitions, corresponding to the time required for a 0.1 °C decrease in temperature, was 5.27 s, with a standard deviation of 0.98 s. The first half of the measurements (first 23 intervals) proceeded slightly faster than the second half: the average interval for the first half was 4.78 s (standard deviation 0.99 s), while for the second half it was 5.77 s (standard deviation 0.68 s). This behavior is consistent with expectations, as the temperature of the target approaches room temperature, slowing the rate of cooling. Throughout the cooling process, the phantom was left completely undisturbed to ensure the repeatability of the measurements. The collected scattering data were later used to perform microwave image reconstruction at each temperature step, allowing us to identify the temperature threshold at which the target region becomes distinguishable—an indicator of the system’s sensitivity to dielectric contrasts comparable to those expected in early Parkinson’s disease.

## 5. Results and Discussion

**Figure 11:**
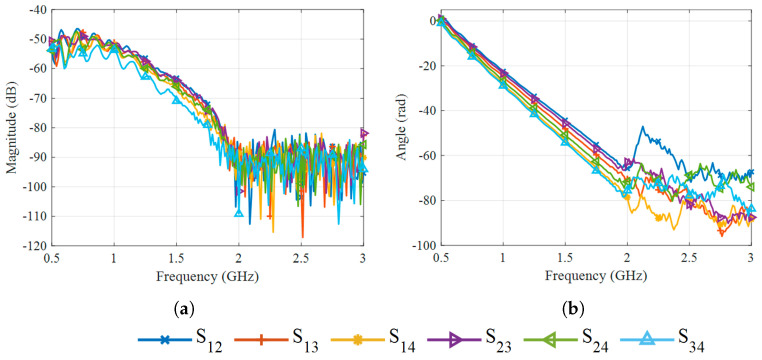
Frequency-domain analysis of the differential transmission scattering parameters. Information becomes unreliable above 2 GHz, leading to the selection of the 0.5–2 GHz range for imaging. (**a**) Magnitude. (**b**) Phase.

**Figure 12:**
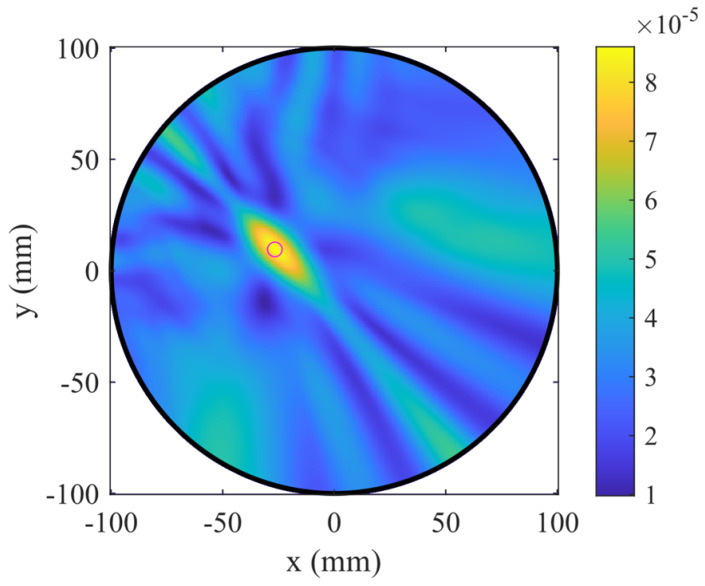
Reconstructed differential image obtained from the comparison between the target filled with air (test case) and with water (background). The target position, indicated with a magenta circle, is correctly localized, confirming the algorithm’s proper operation.

To validate the reconstruction algorithm, a preliminary test was performed using a configuration with a strong dielectric contrast, which represents an easier case to image. Specifically, the differential data were obtained by comparing the situation in which the target tube inside the phantom was filled with air (test case) against the reference case, in which it was filled with water (background). The magnitude and phase of the differential transmission scattering parameters, as shown in Figure 11, were analyzed as a function of frequency, revealing that the information becomes unreliable above 2 GHz. Consequently, the frequency range from 0.5 GHz to 2 GHz, comprising 121 frequency points, was selected for image reconstruction in the subsequent experiments. The reconstructed image, shown in Figure 12, correctly localizes the target at its expected position, confirming the proper functioning of the imaging algorithm. Having established the algorithm’s validity and the reliable frequency band, the following analysis focuses on evaluating the system’s sensitivity to small, controlled dielectric variations induced by temperature changes.

**Figure 13:**
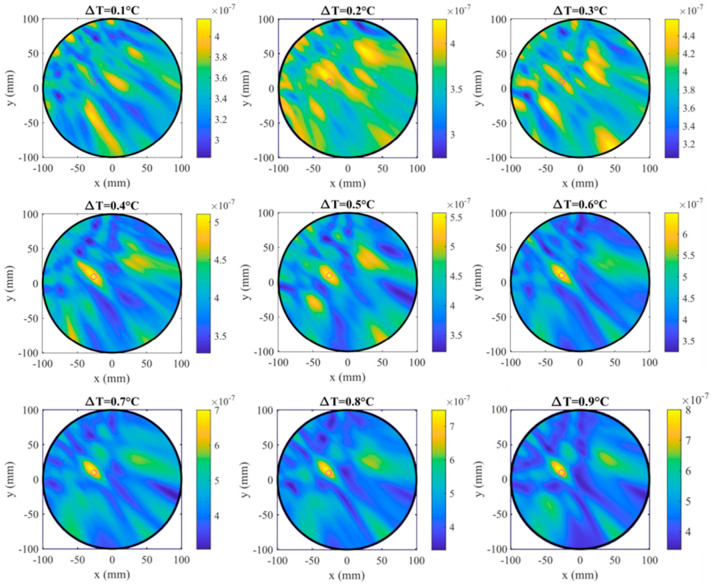
Reconstructed differential images obtained for temperature differences ΔT = 0.1 °C to 0.9 °C. All images display the target position with a magenta circle.

Figure 13 shows the reconstructed images obtained for temperature differences from 0.1 °C to 0.9 °C. For small temperature variations (ΔT ≤ 0.3 °C), the reconstructed field does not exhibit any clearly localized maximum, indicating that such minor dielectric contrasts fall below the system’s sensitivity threshold. Starting from ΔT = 0.4 °C, a distinct intensity peak emerges at the position corresponding to the actual target, demonstrating the ability of the system to detect permittivity variations induced by temperature changes above this limit. As ΔT increases, the reconstructed maximum becomes progressively more pronounced and spatially well localized, confirming the growing dielectric contrast.

**Figure 14:**
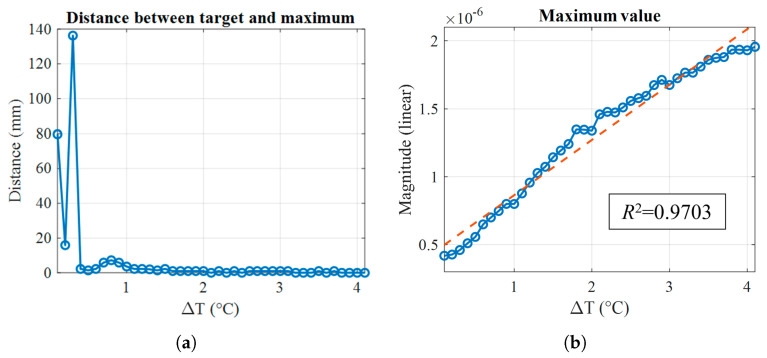
Quantitative analysis of the reconstructed images: (**a**) distance between the reconstructed maximum and the true target position as a function of ΔT; (**b**) linear magnitude of the reconstructed maximum versus ΔT, with the red dashed line showing the fitted linear regression. The coefficient of determination $R^{2}$ is displayed in the inset.

To further quantify the system’s response, Figure 14 reports two metrics derived from the reconstructed images: in Figure 14a, the distance between the actual target barycenter position and the reconstructed maximum; and in Figure 14b, the magnitude of the reconstructed maximum as a function of ΔT. The first plot confirms that the reconstructed maximum becomes spatially accurate for ΔT ≥ 0.4 °C, while the second shows that its amplitude exhibits a linear increase with ΔT. This linear trend suggests that the reconstructed amplitude scales proportionally with the dielectric contrast, supporting the system’s potential for quantitative monitoring of changes in permittivity.

The results demonstrate that for temperature differences smaller than 0.4 °C, the induced dielectric contrast remains below the system’s detection threshold, yielding diffuse and non-localized images. In contrast, when the temperature difference exceeds 0.4 °C, a clear and spatially accurate maximum appears in the reconstructed maps, revealing the position of the target. To relate this sensitivity threshold to the corresponding dielectric variations, we refer to Table 1 and Table 2 where, for deionized water, the equivalent changes in permittivity and conductivity are associated with each temperature step. Specifically, a temperature difference of 0.4 °C corresponds to a relative variation of approximately 0.17% in $\varepsilon_{r}$ and 0.0047% in σ. Furthermore, the linear dependence of the amplitude of the reconstructed maximum with respect to the temperature variation (see Figure 14b) can be quantified through the coefficient of determination $R^{2}$. The obtained value is $R^{2}=0.9703$, indicating that the data are highly consistent with a linear trend. This result confirms that the imaging response scales proportionally with the underlying permittivity change. This behavior provides experimental evidence that the proposed differential processing approach can reliably quantify small dielectric perturbations, thus validating the concept of temperature-controlled dielectric modulation as an effective strategy for calibration and sensitivity assessment in microwave sensing.

## 6. Conclusions and Perspectives

In this work, we investigated the feasibility of MWI for the early detection of Parkinson’s disease. To this end, an MWI system composed of four antennas was developed and employed in combination with the MFBF algorithm for image reconstruction. A dynamic cylindrical phantom was designed and fabricated to experimentally assess the system’s sensitivity to small dielectric variations representative of pathological changes. The phantom exploited the temperature-dependent dielectric properties of water, which were first characterized theoretically and then experimentally validated. The obtained results demonstrate that the system is capable of reliably detecting relative permittivity variations as small as approximately 0.17%.

An additional promising strategy concerns the exploitation of the intrinsic left–right asymmetry of early Parkinson’s disease. Since the pathological dielectric alteration is expected to occur predominantly on one side, the contralateral hemisphere can serve as an internal background reference. This symmetry-based approach enables the extraction of the target response by comparing the electromagnetic fields or reconstructed images of the two hemispheres, thereby avoiding the need for time-differential measurements on the same subject. Implementing such symmetry-based preprocessing within the proposed MFBF framework could enhance detection sensitivity and robustness while simplifying clinical acquisition protocols.

Future developments of this work will focus on increasing the realism of the experimental models by designing anatomically and dielectrically accurate phantoms, and by deepening the understanding of the physiological and dielectric alterations associated with Parkinson’s disease. Ultimately, these efforts aim to move toward a validation phase on human subjects, paving the way for the potential translation of MWI into clinical applications for neurodegenerative disease monitoring.

## Figures and Tables

**Table 1:** Relative sensitivity of σ for 0.1 °C temperature variation at representative frequencies.

Frequency (GHz)	$\eta_{\sigma }$ Debye (%)	$\eta_{\sigma }$ Measured (%)
0.5	$\left(1.6\pm 0.05\right)\times 10^{-4}$	$\left(6.3\pm 0.05\right)\times 10^{-5}$
1.0	$\left(6.4\pm 0.05\right)\times 10^{-4}$	$\left(4.4\pm 0.05\right)\times 10^{-4}$
1.5	$\left(1.4\pm 0.05\right)\times 10^{-3}$	$\left(1.3\pm 0.05\right)\times 10^{-3}$
2.0	$\left(2.5\pm 0.05\right)\times 10^{-3}$	$\left(2.6\pm 0.05\right)\times 10^{-3}$
2.5	$\left(3.9\pm 0.05\right)\times 10^{-3}$	$\left(4.1\pm 0.05\right)\times 10^{-3}$
3.0	$\left(5.6\pm 0.05\right)\times 10^{-3}$	$\left(6.0\pm 0.05\right)\times 10^{-3}$

**Table 2:** Relative sensitivity of $\varepsilon_{r}$ for 0.1 °C temperature variation at representative frequencies.

Frequency (GHz)	$\eta_{\varepsilon_{r}}$ Debye (%)	$\eta_{\varepsilon_{r}}$ Measured (%)
0.5	$\left(4.4\pm 0.05\right)\times 10^{-2}$	$\left(4.9\pm 0.05\right)\times 10^{-2}$
1.0	$\left(4.3\pm 0.05\right)\times 10^{-2}$	$\left(4.9\pm 0.05\right)\times 10^{-2}$
1.5	$\left(4.2\pm 0.05\right)\times 10^{-2}$	$\left(4.5\pm 0.05\right)\times 10^{-2}$
2.0	$\left(4.0\pm 0.05\right)\times 10^{-2}$	$\left(4.6\pm 0.05\right)\times 10^{-2}$
2.5	$\left(3.9\pm 0.05\right)\times 10^{-2}$	$\left(4.4\pm 0.05\right)\times 10^{-2}$
3.0	$\left(3.6\pm 0.05\right)\times 10^{-2}$	$\left(4.1\pm 0.05\right)\times 10^{-2}$

## Data Availability

The original contributions presented in this study are included in the article. The collected dataset used to produce the images is available for download in the Supplementary Materials. Further inquiries can be directed to the corresponding author.

## Supplementary Materials

The following supporting information can be downloaded at: https://www.mdpi.com/article/10.3390/s25247562/s1. The dataset collected and used to produce the results in this work.

## Abbreviations

The following abbreviations are used in this manuscript:

CSF	Cerebrospinal fluid
DaT-SPECT	Dopamine transporter single-photon emission computed tomography
EGRH	Extended gap ridge horn
FDM	Fused deposition modeling
IMUs	Inertial measurement units
MFBF	Multi-frequency bi-focusing
MRI	Magnetic resonance imaging
MWI	Microwave imaging
NfL	Neurofilament light chain
PD	Parkinson’s disease
PET	Photon emission tomography
PLA	Polylactic acid
ROI	Region of interest
SNpc	Substantia nigra pars compacta
SNR	Signal-to-noise ratio
SWR	Standing wave ratio
VNA	Vector network analyzer

## References

[1] Yamashita K.Y., Bhoopatiraju S., Silverglate B.D., Grossberg G.T. (2023). Biomarkers in Parkinson’s disease: A state of the art review. Biomark. Neuropsychiatry.

[2] Zarkali A., Thomas G.E., Zetterberg H., Weil R.S. (2024). Neuroimaging and fluid biomarkers in Parkinson’s disease in an era of targeted interventions. Nat. Commun..

[3] Aubignat M., Tir M., Krystkowiak P. (2021). Non-motor symptoms of Parkinson’s disease from pathophysiology to early diagnosis. Rev. Méd. Interne.

[4] Demailly A., Moreau C., Devos D. (2024). Effectiveness of continuous dopaminergic therapies in Parkinson’s disease: A review of L-DOPA pharmacokinetics/pharmacodynamics. J. Park. Dis..

[5] Regensburger M., Ip C.W., Kohl Z., Schrader C., Urban P.P., Kassubek J., Jost W.H. (2023). Clinical benefit of MAO-B and COMT inhibition in Parkinson’s disease: Practical considerations. J. Neural Transm..

[6] Hacker M.L., Meystedt J.C., Turchan M., Cannard K.R., Harper K., Fan R., Ye F., Davis T.L., Konrad P.E., Charles D. (2023). Eleven-year outcomes of deep brain stimulation in early-stage parkinson disease. Neuromodul. Technol. Neural Interface.

[7] Frank C., Chiu R., Lee J. (2023). Parkinson disease primer, part 2: Management of motor and nonmotor symptoms. Can. Fam. Physician.

[8] Goetz C.G., Tilley B.C., Shaftman S.R., Stebbins G.T., Fahn S., Martinez-Martin P., Poewe W., Sampaio C., Stern M.B., Dodel R. (2008). Movement Disorder Society-sponsored revision of the Unified Parkinson’s Disease Rating Scale (MDS-UPDRS): Scale presentation and clinimetric testing results. Mov. Disord. Off. J. Mov. Disord. Soc..

[9] Tolosa E., Garrido A., Scholz S.W., Poewe W. (2021). Challenges in the diagnosis of Parkinson’s disease. Lancet Neurol..

[10] Saeed U., Lang A.E., Masellis M. (2020). Neuroimaging advances in Parkinson’s disease and atypical Parkinsonian syndromes. Front. Neurol..

[11] Gujral J., Gandhi O.H., Singh S.B., Ahmed M., Ayubcha C., Werner T.J., Revheim M.E., Alavi A. (2024). PET, SPECT, and MRI imaging for evaluation of Parkinson’s disease. Am. J. Nucl. Med. Mol. Imaging.

[12] Burade A., Dagher R., Kaviani P., Lakhani D.A., Sair H.I., Luna L.P. (2025). Comparative Diagnostic Efficacy of Neuromelanin MRI vs. Dopamine Transporter (DAT) imaging in Parkinson’s disease: A Systematic Review. Park. Relat. Disord..

[13] Andersen A.D., Binzer M., Stenager E., Gramsbergen J.B. (2017). Cerebrospinal fluid biomarkers for P arkinson’s disease—A systematic review. Acta Neurol. Scand..

[14] Lin W.C., Lu C.H., Chiu P.Y., Yang S.Y. (2020). Plasma total *α*-synuclein and neurofilament light chain: Clinical validation for discriminating Parkinson’s disease from normal control. Dement. Geriatr. Cogn. Disord..

[15] Quadalti C., Calandra-Buonaura G., Baiardi S., Mastrangelo A., Rossi M., Zenesini C., Giannini G., Candelise N., Sambati L., Polischi B. (2021). Neurofilament light chain and *α*-synuclein RT-QuIC as differential diagnostic biomarkers in parkinsonisms and related syndromes. npj Park. Dis..

[16] Hällqvist J., Bartl M., Dakna M., Schade S., Garagnani P., Bacalini M.G., Pirazzini C., Bhatia K., Schreglmann S., Xylaki M. (2024). Plasma proteomics identify biomarkers predicting Parkinson’s disease up to 7 years before symptom onset. Nat. Commun..

[17] Peres L.B., Calil B.C., da Silva A.P.S.P.B., Dionísio V.C., Vieira M.F., de Oliveira Andrade A., Pereira A.A. (2021). Discrimination between healthy and patients with Parkinson’s disease from hand resting activity using inertial measurement unit. Biomed. Eng. Online.

[18] Abou L., Peters J., Wong E., Akers R., Dossou M.S., Sosnoff J.J., Rice L.A. (2021). Gait and balance assessments using smartphone applications in Parkinson’s disease: A systematic review. J. Med. Syst..

[19] Iqbal S.M., Mahgoub I., Du E., Leavitt M.A., Asghar W. (2021). Advances in healthcare wearable devices. npj Flex. Electron..

[20] Moreau C., Rouaud T., Grabli D., Benatru I., Remy P., Marques A.R., Drapier S., Mariani L.L., Roze E., Devos D. (2023). Overview on wearable sensors for the management of Parkinson’s disease. npj Park. Dis..

[21] He N., Chen Y., LeWitt P.A., Yan F., Haacke E.M. (2023). Application of neuromelanin MR imaging in Parkinson disease. J. Magn. Reson. Imaging.

[22] Shang S., Li D., Tian Y., Li R., Zhao H., Zheng L., Zhang Y., Chen Y.C., Yin X. (2021). Hybrid PET-MRI for early detection of dopaminergic dysfunction and microstructural degradation involved in Parkinson’s disease. Commun. Biol..

[23] Akdemir Ü.Ö., Bora H.A.T., Atay L.Ö. (2021). Dopamine transporter spect imaging in Parkinson’s disease and parkinsoniandisorders. Turk. J. Med. Sci..

[24] Fear E., Stuchly M. (2000). Microwave detection of breast cancer. IEEE Trans. Microw. Theory Tech..

[25] Guardiola M., Capdevila S., Romeu J., Jofre L. (2012). 3-D microwave magnitude combined tomography for breast cancer detection using realistic breast models. IEEE Antennas Wirel. Propag. Lett..

[26] Casu M.R., Vacca M., Tobon J.A., Pulimeno A., Sarwar I., Solimene R., Vipiana F. (2017). A COTS-based microwave imaging system for breast-cancer detection. IEEE Trans. Biomed. Circuits Syst..

[27] Aldhaeebi M.A., Alzoubi K., Almoneef T.S., Bamatraf S.M., Attia H., Ramahi O.M. (2020). Review of microwaves techniques for breast cancer detection. Sensors.

[28] Rana S.P., Dey M., Loretoni R., Duranti M., Ghavami M., Dudley S., Tiberi G. (2023). Radiation-free microwave technology for breast lesion detection using supervised machine learning model. Tomography.

[29] Fhager A., Candefjord S., Elam M., Persson M. (2018). Microwave diagnostics ahead: Saving time and the lives of trauma and stroke patients. IEEE Microw. Mag..

[30] Rodriguez-Duarte D.O., Origlia C., Vasquez J.A.T., Scapaticci R., Crocco L., Vipiana F. (2022). Experimental assessment of real-time brain stroke monitoring via a microwave imaging scanner. IEEE Open J. Antennas Propag..

[31] Guo L., Alqadami A.S.M., Abbosh A. (2023). Stroke Diagnosis Using Microwave Techniques: Review of Systems and Algorithms. IEEE J. Electromagn. Microwaves Med. Biol..

[32] Inum R., Rana M.M., Shushama K.N., Quader M.A. (2018). EBG based microstrip patch antenna for brain tumor detection via scattering parameters in microwave imaging system. Int. J. Biomed. Imaging.

[33] Hossain A., Islam M.T., Beng G.K., Kashem S.B.A., Soliman M.S., Misran N., Chowdhury M.E. (2022). Microwave brain imaging system to detect brain tumor using metamaterial loaded stacked antenna array. Sci. Rep..

[34] Ullah R., Dong Y., Arslan T., Chandran S. (2023). A Machine Learning-Based Classification Method for Monitoring Alzheimer’s Disease Using Electromagnetic Radar Data. IEEE Trans. Microw. Theory Tech..

[35] Farhatullah, Chen X., Zeng D., Ullah R., Nawaz R., Xu J., Arslan T. (2024). A deep learning approach for non-invasive Alzheimer’s monitoring using microwave radar data. Neural Netw..

[36] Cardinali L., Mariano V., Rodriguez-Duarte D.O., Tobón Vasquez J.A., Scapaticci R., Crocco L., Vipiana F. (2025). Early Detection of Alzheimer’s Disease via Machine Learning-Based Microwave Sensing: An Experimental Validation. Sensors.

[37] Akazzim Y., Arias C.P., Jofre M., Mrabet O.E., Romeu J., Jofre-Roca L. (2023). UWB microwave functional brain activity extraction for Parkinson’s disease monitoring. IEEE Sensors J..

[38] Origlia C., Rodriguez-Duarte D.O., Tobon Vasquez J.A., Bolomey J.C., Vipiana F. (2024). Review of microwave near-field sensing and imaging devices in medical applications. Sensors.

[39] Razzicchia E., Lu P., Guo W., Karadima O., Sotiriou I., Ghavami N., Kallos E., Palikaras G., Kosmas P. (2021). Metasurface-Enhanced Antennas for Microwave Brain Imaging. Diagnostics.

[40] Bolomey J.C., Jofre L., Peronnet G. (2003). On the possible use of microwave-active imaging for remote thermal sensing. IEEE Trans. Microw. Theory Tech..

[41] Cardinali L., Aldana R., Rodriguez-Duarte D.O., Tobon-Vasquez J.A., Vipiana F., Jofre-Roca L. Differential Permittivity Modeling in Biological Phantoms via Water Temperature Control. Proceedings of the 2025 IEEE International Conference on Electromagnetics in Advanced Applications (ICEAA).

[42] Kaatze U. (1989). Complex permittivity of water as a function of frequency and temperature. J. Chem. Eng. Data.

[43] Di Bucchianico A. (2008). Coefficient of determination (R 2). Encyclopedia of Statistics in Quality and Reliability.

[44] Keysight Technologies N9918A FieldFox Handheld Microwave Analyzer, 26.5 GHz. https://www.keysight.com/us/en/product/N9918A/fieldfox-a-handheldmicrowave-analyzer-26-5-ghz.html.

[45] Keysight Technologies (2022). N1500A Materials Measurement Suite. https://www.keysight.com/it/en/product/N1500A/materialsmeasurement-suite.html.

[46] Akazzim Y., El Mrabet O., Romeu J., Jofre-Roca L. (2022). Multi-element UWB probe optimization for medical microwave imaging. Sensors.

[47] Akazzim Y., Jofre M., El Mrabet O., Romeu J., Jofre-Roca L. (2023). UWB-modulated microwave imaging for human brain functional monitoring. Sensors.

[48] Rohde & Schwarz. R&S ZNA26 Vector Network Analyzer. https://www.rohde-schwarz.com/us/products/test-and-measurement/network-analyzers/rs-zna-vector-network-analyzers_63493-551810.html.

[49] Rashid S., Jofre L., Garrido A., Gonzalez G., Ding Y., Aguasca A., O’Callaghan J., Romeu J. (2019). 3-D printed UWB microwave bodyscope for biomedical measurements. IEEE Antennas Wirel. Propag. Lett..

[50] Jofre L., Broquetas A., Romeu J., Blanch S., Toda A.P., Fabregas X., Cardama A. (2009). UWB tomographic radar imaging of penetrable and impenetrable objects. Proc. IEEE.

[51] Sonne J., Reddy V., Beato M.R. (2024). Neuroanatomy, substantia nigra. StatPearls [Internet].

